# New Tongue Holder

**Published:** 1851-01

**Authors:** 


*New Tongue Holder.*—The use of such an instrument, in many operations on the mouth and the organs contained within it, is often indispensable to expedition and perfection. In cauterising the upper part of the œsophagus in chronic inflammation, also in tonsilitis, elongated uvula, and in many other diseases to which the posterior mouth is subject, requiring surgical interference, the tongue offers much impediment from its involuntary movements and unmanageableness. Likewise, in many dental operations, it presents considerable difficulty to complete such with safety and comfort to the patient. Many instruments have, from time to time, been invented to overcome this unruly member, but with various and incomplete success, from the fact that none, as yet, have been adopted by general use, and few have stood the test from others than their inventors. Mr. Jamet, dentist, of Baltimore, has recently invented an instrument for this purpose, which has certainly much to recommend it, in its simplicity and neatness, being formed of a very thin steel spring, three-eighths of an inch in width, and perhaps six inches in length, so bent as to press upon the tongue and underneath the chin, fitting the irregularities, passing from within the mouth, over and under the chin. We have used it in several instances, much to our satisfaction, and doubt not it would give equal pleasure to all who will try it.

